# Performance of a parasitic plant and its effects on hosts depends on the interactions between parasite seed family and host species

**DOI:** 10.1093/aobpla/plac063

**Published:** 2022-12-15

**Authors:** Belén Moncalvillo, Diethart Matthies

**Affiliations:** Plant Ecology, Department of Biology, Philipps-Universität Marburg, Marburg 35043, Germany; Plant Ecology, Department of Biology, Philipps-Universität Marburg, Marburg 35043, Germany

**Keywords:** Genetic correlation, genotype by environment interactions, grasslands, legumes, morphological variation, plant–plant interactions, trade-off

## Abstract

Root hemiparasitic plants act as keystone species influencing plant community composition through their differential suppression of host species. Their own performance also strongly depends on interactions with host species. However, little is known about the roles of parasite genetic variation vs. plasticity in these interactions. We grew plants from eight maternal families of the root hemiparasite *Rhinanthus alectorolophus* with six potential host species (two grasses, two legumes and two forbs) and without a host and measured fitness-related and morphological traits of the parasite, host biomass and overall productivity. Parasite biomass and other traits showed strong plastic variation in response to different host species, but were also affected by parasite maternal family. Parasite seed families responded differently to the hosts, indicating genetic variation that could serve as the basis for adaptation to different host plants. However, there were no negative correlations in the performance of families across different hosts, indicating that *R. alectorolophus* has plastic generalist genotypes and is not constrained in its use of different host species by trade-offs in performance. Parasite effects on host biomass (which may indicate virulence) and total productivity (host + parasite biomass) depended on the specific combination of parasite family and host species. Mean biomass of hosts with a parasite family and mean biomass of that family tended to be negatively correlated, suggesting selection for maximum resource extraction from the hosts. Specialization of generalist root hemiparasites may be restricted by a lack of trade-offs in performance across hosts, together with strong spatial and temporal variation in host species availability. The genetic variation in the effects on different hosts highlights the importance of genetic diversity of hemiparasites for their effects on plant community structure and productivity and for the success of using them to restore grassland diversity.

## Teaser

Root hemiparasites have green leaves but attack the roots of other plants and extract water and nutrients from them. Offspring of different mother plants of the root hemiparasite Rhinanthus alectorolophus varied in their response to several host plants and their damage to the hosts also depended on the specific combination of parasite family and host species. Our results suggest that genetic variation within populations of hemiparasites influences the interactions with their hosts. Genetic variation of hemiparasites will be important for their use to reduce productivity in the management of grasslands for conservation.

## Introduction

Parasitic flowering plants represent c. 1 % of angiosperms (c. 4500 species, Heide-Jørgensen 2008). These species attach to the shoots or roots of their host plants via special organs (haustoria) and extract water, nutrients and carbon compounds from them (Těšitel *et al*. 2010). Many parasitic plants are hemiparasites which have green leaves and maintain the ability to photosynthesize (Phoenix and Press 2005). These species are generalist parasites which can grow with a wide range of species as hosts (Hautier *et al*. 2010; Matthies 2017), but their biomass (Matthies 2021), morphology (Campion-Bourget 1982; Jonstrup *et al*. 2016), patterns of allometry (Matthies 1995) and reproduction (Seel *et al*. 1993; Matthies 2017) can vary enormously depending on the host species. While some plant species are resistant against parasite infection and the growth of parasites with them is worse than without a host (Atsatt and Strong 1970; Cameron *et al*. 2006), others increase parasite performance strongly in comparison to autotrophic growth (Seel *et al*. 1993; Matthies 1995).

The interactions between parasitic plants and their hosts show many parallels with those between herbivorous insects and their host plants (Atsatt 1977; Pennings and Callaway 2002). However, while hemiparasites often have broad host ranges, many insects are specialist feeders on one or a few plant species (Jaenike 1990; Forister *et al*. 2015). A common explanation for this specialization of insects is the ‘the jack of all trades is the master of none’ principle, which states that specialists should outperform generalists on any specific host and that selection should therefore favour host specialization (Futuyma and Moreno 1988; Jaenike 1990; García-Robledo and Horvitz 2011). If there is a cost of adaptation to a specific host, a genotype adapted to one host is predicted to be less able to take advantage of another host species due to antagonistic pleiotropy. There should thus be trade-offs in the performance of herbivores with different hosts and a genotype that is well suited to one type of host plant is expected to have a poor performance on another host species, expressed as negative genetic correlations (Futuyma and Moreno 1988). However, empirical tests of such trade-offs in cross-host performance have found them only in some plant–herbivore systems (Henry *et al*. 2008; Gompert and Messina 2016), but not in others (Forister *et al*. 2007; Agosta and Klemens 2009).

While the genetics of the interactions between herbivorous insects and their host plants have been intensively studied (Forister *et al*. 2012), few studies have investigated the genetics of hemiparasite–host interactions (but see Mutikainen *et al*. 2000; Koskela *et al*. 2002; Ahonen *et al*. 2006). Individuals from different populations of hemiparasites grown in a common environment have been found to differ in a variety of traits (Zopfi 1993; Mutikainen *et al*. 2000; Rowntree *et al*. 2011). Some of this genetic variation in traits is likely to represent adaptation to different environmental conditions, e.g. habitat management (Zopfi 1993). Hemiparasite performance with individual host species has also been shown to differ among parasite populations and maternal families (Campion-Bourget 1982; Jonstrup *et al*. 2016; Sandner and Matthies 2017), indicating genetic variability in parasite responses to different host species, a precondition for the evolution of genotypes adapted to different hosts. However, the single study that has investigated possible trade-offs in the performance of hemiparasite genotypes across different hosts (Ahonen *et al*. 2006) did not find negative correlations between the biomass produced by the parasite *Rhinanthus angustifolius* with the grass *Agrostis capillaris* and the legume *Trifolium pratense*.

Root hemiparasites can have strong negative effects on their hosts (Ameloot *et al*. 2005; Phoenix and Press 2005; Press and Phoenix 2005; Matthies 2017). As host species vary in their sensitivity to hemiparasites, the parasites can change the competitive balance between plant species (Matthies 1996; Demey *et al*. 2015; Rowntree and Craig 2019) and act as ecosystem engineers. They often also reduce total productivity of the community (Davies *et al*. 1997; Mudrák and Lepš 2010; Borowicz *et al*. 2019). The outcome of the infection depends on the genetics of both parasites and hosts, as the impact of parasitism may differ between parasite populations (Mutikainen *et al*. 2000; Sandner and Matthies 2017) and seed families (Ahonen *et al*. 2006), and also depend on host genotype (Rowntree *et al*. 2011). However, it is not known whether hemiparasite seed families that benefit most from a host also cause the greatest damage to it, i.e. are most virulent.

To investigate the role of genetic variation in the interactions between a root hemiparasite and its hosts, we grew eight maternal families from a single population of the hemiparasite *Rhinanthus alectorolophus* with six potential host species (two grasses, two legumes and two forbs) in a common environment. Since *R. alectorolophus* is capable of autotrophic growth (Sandner and Matthies 2017), we also grew all parasite families without a host for comparison and to test for correlations between autotrophic and heterotrophic performance. A positive correlation between autotrophic and heterotrophic growth of seed families would suggest that some parasite seed families are generally more vigorous than others, while a negative relationship would suggest that an improved ability to extract resources from a host reduces the ability to grow autotrophically, increasing the degree of dependence on the host (Ahonen *et al*. 2006). The variation among families will indicate the evolutionary potential of the studied hemiparasite population, while the results of studies on the differentiation among hemiparasite populations (Campion-Bourget 1982; Mutikainen *et al*. 2000; Jonstrup *et al*. 2016; Sandner and Matthies 2017) reflected past genetic processes. Our study included a wider range of host species (six instead of two) than the related study by Ahonen *et al*. (2006), allowing us a more general test of the role of variation among families in the interactions between hemiparasites and hosts. The use of more hosts also allowed us to investigate whether those host families that are most beneficial for the parasites are also those that produce the least biomass with the parasites. However, due to restrictions on glasshouse space we had to use fewer parasitic families (8 instead of 25) than Ahonen *et al*. (2006).

We measured fitness-related traits and morphological characters of the parasite that have been used to delimit infraspecific taxa and ecotypes, host biomass and total productivity. We addressed the following specific questions: (i) Are traits of the parasite influenced by host species and maternal family, and do maternal families of *R. alectorolophus* react differently to individual host species? (ii) Are there trade-offs between the performance of parasite families with different hosts? (iii) Are the effects of *R. alectorolophus* on host biomass and total productivity influenced by parasite maternal family and are the negative effects of the different parasite families on the hosts and the performance of those families correlated?

## Materials and Methods

### Study species

*Rhinanthus alectorolophus* (Orobanchaceae) is an annual facultative hemiparasite growing up to 80 cm. It is usually pollinated by insects, but selfing is possible (Sandner and Matthies 2017). *Rhinanthus alectorolophus* is commonly found in Central European grasslands, especially in areas of low productivity and high light availability (Těšitel *et al*. 2015), and was in former times also a weed of cereal crops (Hartl 1974). It is one of the most frequently used species for studies on the ecology of hemiparasites, together with *R. minor* and *R. angustifolius* (Těšitel *et al*. 2011).

As host plants we selected two grasses (*Dactylis glomerata* and *Lolium perenne*), two legumes (*Medicago sativa* and *Trifolium repens*) and two non-leguminous forbs (*Sanguisorba minor* and *Sinapis alba*), which will be referred to by their genus names in the following. All these species occur together with *R. alectorolophus*.

### The experiment

Seeds of *R. alectorolophus* were collected in a large population in northern Hessen (Germany) from eight large mother plants that were separated by at least 50 m. Seeds from the same mother plant (seed families) are at least half-sibs, but could also be full-sibs or be even more closely related because they resulted from self-fertilization. To break dormancy, the seeds were placed on moist filter paper in Petri dishes and kept at 5 °C until cotyledons had formed. Seeds of the host species were obtained from a commercial supplier (Appels Wilde Samen, Darmstadt, Germany) and germinated in Petri dishes at room temperature. Two seedlings of each host species were planted into 48 pots (11 cm × 11 cm) filled with a 4:1 mixture of commercial potting soil (TKS, Floragard, Oldenburg, Germany) and sand. After 2 weeks of growth, one seedling of the parasite was planted into each pot. For each combination of parasite maternal family and host species eight replicates were set up. In addition, five parasites from each family were planted into pots without a host. Plants were kept in a growth chamber at 16 h of light by metal halide lamps and a 20 °C/15 °C (day/night) temperature regime. Pots were watered regularly and their position randomized every 2 weeks. To follow the development of the parasites, the length of their longest leaf was measured after 2 and 4 weeks of growth and the day when a parasite produced its first flower was recorded.

After 9 weeks of growth, when the parasites were well developed and all but the smallest were flowering or fruiting, several traits were measured for each parasite: the height of each plant, the length of the vegetative part of the stem, total branch length as the sum of the length of all branches plus the height of a plant, the number of nodes below the lowermost flower (vegetative nodes), the length of the first five internodes, the number of flowers on the main inflorescence, the total number of flowers, the length of a fully developed flower, the diameter of a ripe fruit and the length and width of the longest leaf. Parasite leaf chlorophyll content was measured with a portable chlorophyll meter (SPAD-502, Minolta, Osaka, Japan) and the values obtained transformed into actual chlorophyll concentrations using the formula for total chlorophyll content given by Richardson *et al*. (2002). Parasites and hosts were harvested separately above ground, and all plant material dried for 48 h at 80°C and weighed.

### Statistical analyses

Effects of host treatment and parasite family on parasite traits were studied by two-factor ANOVAs. We considered host treatment as a fixed factor and parasite maternal family as a random factor. According to the rules for mixed models (Zar 2010), the effect of host treatment was tested against the host by family interaction, while the other effects were tested against the residual variation. Parasite biomass, total branch length, the length of the first five internodes and the number of flowers were log-transformed to obtain normally distributed residuals. Simple main effects were calculated to test family effects within each host treatment. To investigate whether host species identity and maternal family influenced the allometric relationships between traits of the parasite and its biomass, we tested the effects of host and family adjusted for the effects of biomass in a general linear model that included the two factors and (log) parasite biomass as a covariate. The effects of biomass, host and the biomass by host interaction were tested against the interactions of these effects with family, while all other effects were tested against the residual.

To test if *R. alectorolophus* showed trade-offs in its performance with different host species or grown autotrophically, we related the mean biomass of the individuals of each parasite family when grown with a host to that produced with each of the other host species, and to that grown without a host. We studied the effects of host species and parasite maternal family on host biomass and total productivity (parasite + host biomass) by two-factor ANOVAs. To test for a possible relationship between parasite damage to a host and its performance, we related in a general linear model the mean biomass of each host grown with each parasite family to the mean biomass of that family grown with that host, after adjusting for the overall host effect. We presented this relationship in a partial residual plot created using the R-package *visreg* (Breheny and Burchett 2017). The relationship between the biomass of the individual parasites and that of their hosts was analysed by regressing the biomass of the host plant in each pot on that of the parasite separately for each host species. Statistical analyses were carried out with R 4.1.1 (R Core Team 2021).

## Results

### Effects of host and maternal family on parasite biomass

**Figure 1.**
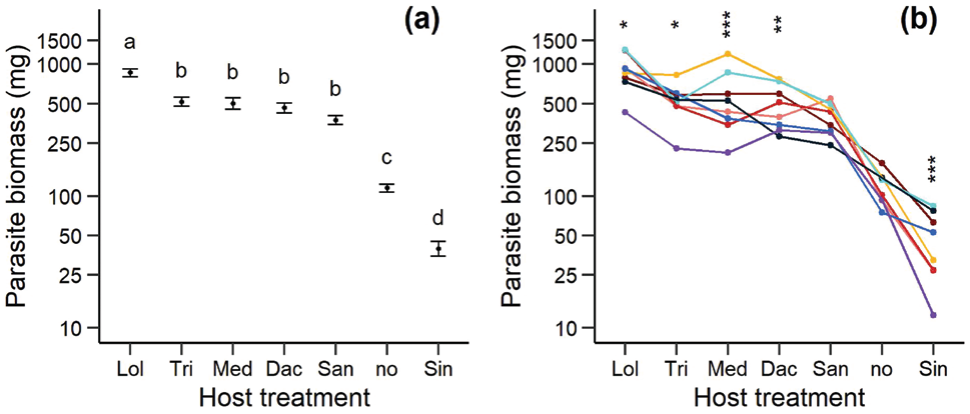
(A) Mean biomass of the parasite *R. alectorolophus* grown with six different host species and without a host (Lol, *Lolium perenne*; Tri, *Trifolium repens*; Med, *Medicago sativa*; Dac, *Dactylis glomerata*; San, *Sanguisorba minor*; no, no host; Sin, *Sinapis alba*). Different letters indicate significant differences at the 0.05 level (Tukey test). Vertical lines show ±1 SE. (B) Reaction norms of the biomass of eight maternal families of the parasite in response to the host treatments. Hosts are in the order of decreasing mean parasite biomass. Significant differences among families within a host treatment: *P < 0.05; **P < 0.01; ***P < 0.001. Note log-scale for biomass.

The growth of *R. alectorolophus* as measured by the length of its longest leaf differed among the host treatments already after 2 weeks (*F*_6, 398_ = 15.24, *P* < 0.001; **see****Supporting Information—Fig. S1**) and these differences became more pronounced over time. The biomass of the parasite *R. alectorolophus* at the end of the experiment was also strongly influenced by the host treatment (Table 1). *Lolium* was the best host, followed by a group of species that were of a lesser quality as hosts (*Trifolium*, *Medicago*, *Dactylis* and *Sanguisorba*; Fig. 1A). The biomass of *R. alectorolophus* grown without a host was much lower, and that of parasites grown with *Sinapis* was even lower than without a host. The size of parasites grown with the best host *Lolium* was 21 times that of the size grown with the worst host *Sinapis.* The type of host treatment influenced parasite biomass even if the data for the very poor host *Sinapis* and for parasites growing without a host were omitted from the analysis (*F*_4, 28_ = 10.7, *P* < 0.001).

**Table 1.** Effect of host treatment, parasite maternal family and their interaction on traits of the hemiparasite *R. alectorolophus.* df_res_ = 331–349. *P*-values <0.05 are in bold face.

Trait	Host species (df = 6)		Family (df = 7)		Host × Family (df = 42)	
	*F*	*P*	*F*	*P*	*F*	*P*
Parasite biomass (log)	86.8	**<0.001**	12.5	**<0.001**	2.1	**<0.001**
Height	75.8	**<0.001**	10.8	**<0.001**	2.1	**<0.001**
Length of vegetative part of stem	63.6	**<0.001**	10.6	**<0.001**	2.2	**<0.001**
Total branch length (log)	76.6	**<0.001**	8.5	**<0.001**	2.1	**<0.001**
Number of vegetative nodes	2.3	0.057	53.4	**<0.001**	1.5	**0.035**
Length of first five internodes (log)	21.0	**<0.001**	13.4	**<0.001**	1.7	**0.005**
Days until flowering	19.9	**<0.001**	22.9	**<0.001**	1.8	**0.004**
Number of flowers (log)	65.1	**<0.001**	8.2	**<0.001**	2.0	**<0.001**
Number of flowers at main inflorescence (log)	67.4	**<0.001**	8.7	**<0.001**	2.1	**<0.001**
Flower length	39.4	**<0.001**	6.1	**<0.001**	0.9	0.731
Diameter of ripe fruit	33.5	**<0.001**	4.3	**<0.001**	1.8	**0.004**
Leaf chlorophyll content	36.9	**<0.001**	4.2	**<0.001**	2.2	**<0.001**
Length of longest leaf	67.5	**<0.001**	14.9	**<0.001**	2.1	**<0.001**
Width of longest leaf	80.6	**<0.001**	8.8	**<0.001**	1.4	0.057

The mean biomass of the parasites from the eight maternal families varied strongly and there was a significant interaction between the effects of host treatment and parasite family, i.e. the parasites from the individual families responded differently to the host treatments and the reaction norms of the different families crossed each other (Table 1; Fig. 1B). The analysis of simple main effects showed that parasite families grown with *Lolium*, *Trifolium*, *Medicago Dactylis* and *Sinapis* differed significantly in their biomass, while the variation among families grown with *Sanguisorba* or without a host was very small. Omitting the two worst host treatments did not change the results qualitatively (family: *F*_7, 268_ = 11.1, *P* < 0.001; host × family: *F*_28, 268_ = 1.7, *P* = 0.023).

### Effects of host and maternal family on other parasite traits

**Figure 2.**
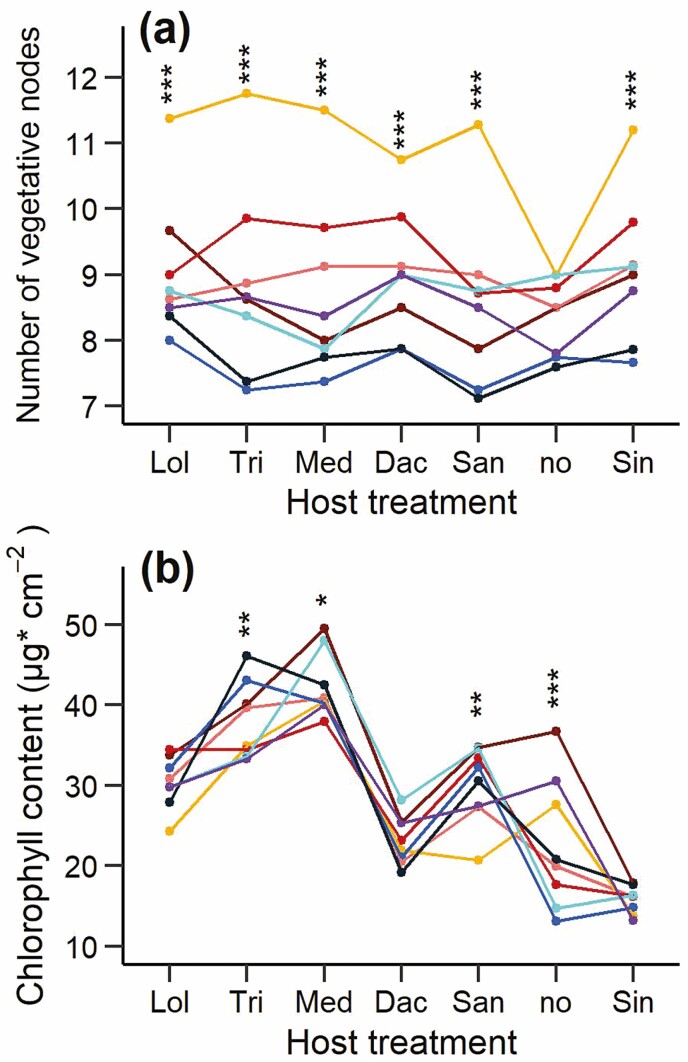
(A) The number of vegetative nodes, and (B) leaf chlorophyll content for eight maternal families of the parasite *R. alectorolophus* grown with six different host species and without a host. The colour of each family is the same as in Fig. 1. Host species are in order of decreasing parasite biomass. Significant differences among families within each host: *P < 0.05; **P < 0.01; ***P < 0.001. For abbreviations of host species names, see Fig. 1.

Both host species and parasite maternal family significantly influenced not only parasite biomass, but also all other measured traits, and in most cases there was a significant interaction between the two factors (Table 1). Most traits were strongly correlated with biomass, i.e. parasite size (Table 2). Thus, similar patterns as for biomass were observed in most other traits in response to host treatment (Table 3) and maternal family **[see****Supporting Information—Fig. S2****]**. The strong correlation between the number of flowers and biomass indicated that biomass is a good proxy for fitness in *R. alectorolophus*. Trait values were usually highest for parasites grown with *Lolium* and lowest for those grown with *Sinapis*. Parasites with *Lolium* grew higher, needed less time to start flowering and produced more flowers and larger flowers and fruits than parasites grown with less beneficial hosts (Table 3). However, the correlations between parasite biomass and leaf chlorophyll content or length of the lowest five internodes were much weaker and there was only a very weak relation to the number of vegetative nodes (Table 2), which was more strongly influenced by maternal family than by host treatment (Table 1; Fig. 2A). Leaf chlorophyll content was highest for parasites grown with the legumes *Medicago* and *Trifolium*, and relatively low not only with *Sinapis* and without a host, but also with the good host *Dactylis* (Fig. 2B). Host treatment and maternal family significantly affected all studied traits of the parasite even after adjusting for differences in biomass, indicating that their effects on the various traits were not simple side effects of their effects on parasite growth, but that both host treatment and maternal family influenced the morphology and architecture of the parasite **[see****Supporting Information—Table S1****]**.

**Table 2.** Correlations between various traits of the hemiparasite *R. alectorolophus* and its biomass. *P*-values <0.05 are in bold face.

Trait	*r*	*P*
Height	0.92	**<0.001**
Length of vegetative part of stem	0.88	**<0.001**
Total branch length (log)	0.95	**<0.001**
Number of vegetative nodes	0.10	0.054
Length of first five internodes (log)	0.51	**<0.001**
Days until flowering	−0.58	**<0.001**
Number of flowers at main inflorescence (log)	0.93	**<0.001**
Number of flowers (log)	0.93	**<0.001**
Flower length	0.65	**<0.001**
Diameter of ripe fruit	0.79	**<0.001**
Leaf chlorophyll content	0.55	**<0.001**
Length of longest leaf	0.93	**<0.001**
Width of longest leaf	0.87	**<0.001**

**Table 3.** Influence of the host treatment and maternal family on traits of the hemiparasite *R. alectorolophus*. Host species are in order of decreasing mean parasite biomass. For families the range of mean values is given. For abbreviations of host species names, see legend to Fig. 1; for family means, **see****Supporting Information—Table S2**; and for the combination of host and family effects, **see****Supporting Information—Fig. S2**.

Trait	Host							Family	
	Lol	Tri	Med	Dac	San	no	Sin	Min.	Max.
Parasite biomass (mg)^a^	867.0	505.8	502.3	464.5	377.6	114.3	40.0	156.7	421.7
Height (cm)	38.0	33.6	32.9	34.3	29.8	15.3	11.5	23.5	32.5
Length of vegetative part of stem (cm)	27.7	26.3	26.1	26.3	23.3	12.3	10.2	19.4	26.0
Total branch length (cm)^a^	51.1	34.7	33.5	36.0	29.4	15.1	10.1	20.7	32.1
Number of vegetative nodes	9.0	8.8	8.7	9.0	8.6	8.4	9.1	7.6	11.0
Length of first five internodes (cm)^a^	8.2	10.4	9.3	8.9	9.5	6.6	5.9	7.2	10.2
Days until flowering	48.3	49.5	49.0	49.0	49.2	51.5	56.3	47.0	52.8
Number of flowers^a^	15.1	8.0	7.3	8.0	7.9	4.1	1.2	4.1	7.7
Number of flowers at main inflorescence^a^	11.7	7.7	6.8	7.5	7.7	4.1	1.2	4.0	7.5
Flower length (mm)	19.7	19.6	19.4	19.3	18.9	17.4	17.1	18.0	19.3
Diameter of ripe fruit (mm)	13.3	13.1	13.1	12.9	12.0	6.8	6.9	10.0	12.4
Leaf chlorophyll content (µg * cm^−2^)	30.4	38.2	42.5	23.1	30.1	22.6	15.7	26.3	34.0
Length of longest leaf (mm)	49.8	43.2	43.6	41.3	39.8	26.1	14.5	28.3	41.8
Width of longest leaf (mm)	14.4	12.3	12.2	11.3	11.7	7.4	3.7	8.5	11.7

^a^Geometric means.

### Possible trade-offs between the performance of parasite families with different hosts

**Figure 3.**
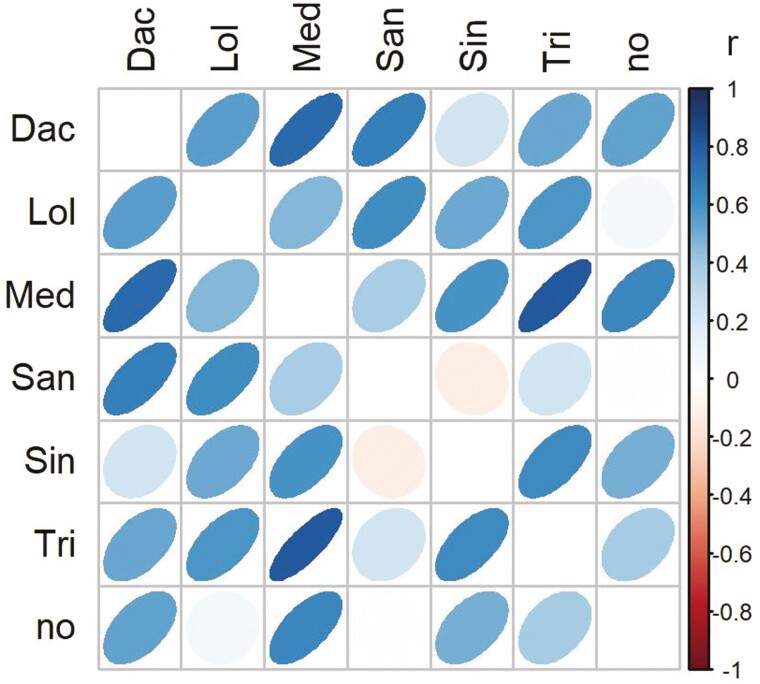
Correlation matrix for the pairwise relationships between the mean biomass of individuals of the hemiparasite *R. alectorolophus* belonging to eight different families grown with different hosts and without a host. Colours indicate correlation coefficients. For abbreviations of host species names, see Fig. 1; for individual data points, **see****Supporting Information—Figs S3****and****S4**.

Negative correlations between the biomass of seed families grown with two host species would indicate trade-offs in the performance with different hosts. However, we did not find trade-offs between the biomass of families when growing with different hosts or without a host (Fig. 3; **see****Supporting Information—Figs S3****and****S4**). Instead, nearly all pairwise relationships were positive, and the two negative ones (Fig. 3, *Sanguisorba* and *Sinapis*, *Sanguisorba* and no host) were very weak (*r* = −0.12, *P* = 0.780; **see****Supporting Information—Fig. S3m**; *r* = −0.02, *P* = 0.967; **see****Supporting Information—Fig. S4d**). The positive relations were strongest between the biomass of the maternal parasite families grown with *Dactylis* and *Medicago* and *Medicago* and *Trifolium* (Fig. 3).

### Host biomass and its relation to parasite biomass

**Figure 4.**
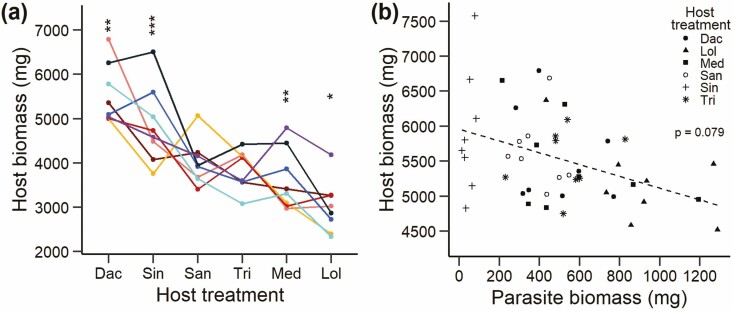
(A) Reaction norms of the biomass of the six host species with eight maternal families of the hemiparasite *R. alectorolophus*. Host species are in order of decreasing host biomass. For abbreviations of host species names, see Fig. 1. Significant differences among families within each host: *P < 0.05; **P < 0.01; ***P < 0.001. The colour code for each family is the same as in Fig. 1. (B) Partial residual plot of the relationship between the mean biomass of each host species grown with each of the eight different seed families of the hemiparasite *R. alectorolophus* and the mean biomass produced by these families with that host, adjusted for the overall effect of individual host species (host species: *F*_5, 41_ = 16.6, *P* < 0.001; parasite biomass: *F*_1, 41_ = 3.2, *P* = 0.079).

**Figure 5.**
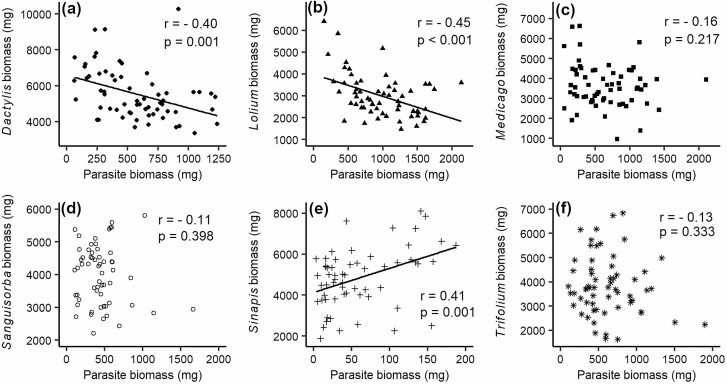
Relationship between the biomass of the host individuals and the individual of the hemiparasite *R. alectorolophus* grown in the same pot. Regression lines are shown if *P* < 0.05. Hosts: (a) *Dactylis glomerata*, (b) *Lolium perenne*, (c) *Medicago sativa*, (d) *Sanguisorba minor*, (e) *Sinapis alba*, (f) *Trifolium repens*.

The biomass of the hosts varied strongly among the six species (*F*_5, 35_ = 17.04, *P* < 0.001), from 3011 mg for *Lolium* to 5540 mg for *Dactylis*, but depended also on parasite family (*F*_7, 320_ = 3.42, *P* < 0.002) and its interaction with host treatment (*F*_35, 320_ = 2.48, *P* < 0.001). Simple main effects showed that parasite family significantly affected the biomass of *Dactylis*, *Lolium*, *Medicago* and *Sinapis*, but not that of *Sanguisorba* and *Trifolium* (Fig. 4A). Total productivity (host + parasite biomass) also depended on host treatment (*F*_5, 35_ = 9.90, *P* < 0.001) and the specific combination of host and parasite family (*F*_35, 320_ = 2.56, *P* < 0.001), while the main effect of family was weak (*F*_7, 320_ = 1.92, *P* = 0.067). The biomass of a host with a certain parasite family tended to be negatively associated with the biomass of that parasite family (*P* = 0.079; Fig. 4B). The biomass of individuals of the two grasses *Dactylis* and *Lolium* decreased significantly with the biomass of the parasite *R. alectorolophus* grown in the same pot (Fig. 5A and B), which can be related to stronger effects of larger parasite individuals on their hosts or to larger hosts exerting stronger competitive effects on the parasite. In contrast, negative but non-significant relationships between host and parasite biomass were found for combinations of *R. alectorolophus* with *Medicago*, *Sanguisorba* or *Trifolium* (Fig. 5C, D and F). The biomass of *Sinapis* and *R. alectorolophus* (Fig. 5E) was positively related, but the parasites growing with *Sinapis* were very small.

## Discussion

### Influence of the host plants on parasite performance

Five of the six host species (the grasses *Lolium* and *Dactylis*, the legumes *Trifolium* and *Medicago*, and the forb *Sanguisorba*) strongly increased growth and reproduction of the hemiparasite *R. alectorolophus* in comparison to that of parasites grown autotrophically, confirming that root hemiparasites like *R. alectorolophus* have a wide host range (Sandner and Matthies 2018; Matthies 2021). However, the performance of *R. alectorolophus* grown with the forb *Sinapis* was worse than that of parasites grown without a host, indicating resistance of *Sinapis* against parasitism or a low quality of the solutes the parasite extracted from this host. Negative effects of unsuitable host species on hemiparasites have been observed in other studies (Atsatt and Strong 1970; Ahonen *et al*. 2006; Cameron *et al*. 2006) and could be due to competition for light, water and nutrients by the host (Matthies 1995; Cameron *et al*. 2005), or related to the costs for the parasite of investing into non-functional haustoria (Ahonen *et al*. 2006).

Grasses and legumes have been found to be generally good hosts for *Rhinanthus* spp. (Seel *et al*. 1993; Matthies 2021), but hosts from each functional group may provide hemiparasites with different compounds (Govier *et al*. 1967). Hemiparasites can obtain large amounts of carbon compounds from grasses (Těšitel *et al*. 2010), while legumes can be especially important for providing nitrogen due to their association with nitrogen-fixing bacteria (Govier *et al*. 1967; Haynes 2021). In our study, the grass *Lolium* was a significantly more beneficial host for *R. alectorolophus* than the other species. The two legumes, although not the best hosts in terms of biomass, resulted in a particularly high chlorophyll content of the parasites, presumably due to their provision of nitrogen. Leaf chlorophyll content of the related parasite *Melampyrum arvense* was strongly related to nitrogen content and higher in parasites grown with legumes than with hosts from other functional groups (Matthies 2017).

### Genetic variation in parasite fitness with different host species

The performance of *R. alectorolophus* was strongly influenced by parasite maternal family, indicating genetic variation in growth and reproduction within the studied population, in contrast to the results of Hautier *et al*. (2010), but in line with among family variation found in *R. angustifolius* (Ahonen *et al*. 2006) and among population variation in other studies (Lammi *et al*. 1999; Mutikainen *et al*. 2000; Jonstrup *et al*. 2016; Sandner and Matthies 2017). However, plants from all the seed families could survive and reproduce successfully on several host plants, suggesting that they have plastic generalist genotypes (García-Robledo and Horvitz 2011).

Other factors apart from genetic variation (both additive and non-additive) could also have contributed to the observed differences among maternal seed families. The maternal environment can affect the quantity and quality of starch reserves, mRNAs, proteins, hormones and other metabolites packaged into seeds (Herman and Sultan 2011). However, these effects of the maternal environment on progeny are largest during the early stages of the development and diminish over time (Roach and Wulff 1987; Herman and Sultan 2011; Auge *et al.* 2017). Late traits like the biomass of adult *R. alectorolophus* are thus less likely to be affected by these maternal effects. Another potential mechanism for effects of the maternal environment on progeny are epigenetic changes, resulting in transgenerational plasticity (Galloway 2005; Herman and Sultan 2011). However, in experiments that found these epigenetic effects they were mostly induced by strong stresses (Hauser *et al.* 2011). In the current experiment, all seeds were sampled from large plants that had apparently not experienced very different stress levels.

The fitness of the parasites depended also on the specific combination of parasite family and host treatment (significant family by host treatment interaction). This variation in the performance of parasite families could serve as the basis for their adaptation to different host species. However, there were no negative correlations between the performance of the parasite families across different host treatment. Instead, we found in nearly all cases positive correlations, because the overall differences among parasite families in performance were strong. Negative genetic correlations would have indicated trade-offs between the fitness of the parasites with different hosts due to antagonistic pleiotropy which are thought to strongly favour host specialization in parasites (Forister *et al.* 2007; García-Robledo and Horvitz 2011). The absence of trade-offs indicates that adaptations increasing the fitness of *R. alectorolophus* with one host species are not likely to reduce its fitness when growing with other host species, reducing the likelihood of specialization to particular hosts. Our results agree with those of Ahonen *et al*. (2006), who found no trade-offs but positive correlations between the performance of *R. angustifolius* across two host species, and several studies on herbivorous insects (e.g. Agosta and Klemens 2009; García-Robledo and Horvitz 2011; Laukkanen *et al*. 2013).

There are several possible reasons for the absence of trade-offs in the performance of *R. alectorolophus* with different hosts and a lack of specialization. The typical habitats of *R. alectorolophus*, like that of most other root hemiparasites, are species-rich and thus for the parasites spatially heterogeneous (Těšitel *et al*. 2015; Holá *et al*. 2017), because the identity of host species available for an individual parasite is largely unpredictable. Such host environments that are variable in space and time favour the evolution of generalists (Futuyma and Moreno 1988; Jaenike 1990; Laukkanen *et al*. 2013). In contrast to many insect herbivores (Forister *et al*. 2015), transmission of root hemiparasites (dispersal) is not directed towards particular host species, which also creates variability in host species over time. If dispersal is not directed towards suitable hosts, specialization incurs high costs in parasites and frequent transmission among hosts is likely to lead to generalist parasites with suboptimal virulence (Rigaud *et al*. 2010). Root hemiparasite individuals can also attack several hosts simultaneously (Gibson and Watkinson 1989; Holá *et al*. 2017) and unspecialized individuals may thus benefit from a mixed diet (Marvier 1998; Sandner and Matthies 2018; but see Matthies 1996), because different host species may provide them with complementary resources (Govier *et al*. 1967; Sandner and Matthies 2018).

It could be interesting to study potential specialization on different hosts for parasite populations that grow in habitats in which single host species are strongly dominant. For example, parasite taxa that formerly grew as weeds of cereals like *R. alectorolophus* ssp. *buccalis*, *R. angustifolius* ssp. *apterus* and *M. arvense* had a far more predictable host environment that may have favoured specialization on a single host species. However, these hemiparasites growing as weeds of crops have strongly declined and have become threatened in many regions (Kornás 1988; Zając and Zając 2014).

There was no trade-off between autotrophic growth of the parasites and their performance with the six hosts, indicating that an increased capability of extracting resources from a host does not lead to a stronger dependency on a host (i.e. no tendency towards holoparasitism). An ability to grow without a host is important for root hemiparasites during the early stages of their life cycle when they have not yet access to a host and also in cases when the available hosts are unsuitable (Atsatt and Strong 1970).

### Effects on other parasite traits

Both the host treatment and maternal family strongly influenced not only the biomass of the parasites, but all other traits. A large part of the variation in nearly all traits could be explained in terms of changes in parasite size, as most traits were strongly correlated with biomass. However, as in other hemiparasites (Matthies 2017), significant effects of host treatment remained even after adjusting for differences in biomass, i.e. parasite individuals of the same biomass but subjected to different host species varied in morphological traits like height, branch length, length of internodes, number and size of flowers, and fruit size. Host plant identity thus also influenced parasite allometry, architecture and morphology. Variation due to host species may partly be responsible for the extensive intraspecific morphological variation commonly observed in root hemiparasites (Zopfi 1993; Pleines *et al*. 2013) and suggests caution when trying to delimit infraspecific taxa on the basis of observational studies (Houston and Wolff 2012; Matthies 2017).

In contrast to other traits, the number of vegetative nodes was only weakly influenced by host treatment and much more strongly by maternal family, confirming that this trait is in hemiparasitic Orobanchaceae less plastic and under stronger genetic control (Campion-Bourget 1982; Zopfi 1993; Jonstrup *et al*. 2016). It is also a trait that varies consistently among seasonal ecotypes (Zopfi 1993). The time to flowering is negatively related to the number of vegetative nodes in *Rhinanthus*, but also influenced by host species (Jonstrup *et al*. 2016; Wesselingh 2016; Matthies 2021). In our experiment, flowering time of *R. alectorolophus* was mainly influenced by seed family, as variation due to the five good host species was very small. Our results support the idea that there is genetic variation within hemiparasitic populations that can allow them to quickly respond to changes in the environment, e.g. due to management, with changes in flowering time, resulting in seasonal ecotypes (Zopfi 1993).

### Genetic variation in the effect of the parasite on the hosts

The biomass of the host plants varied more than 2-fold depending on parasite seed family, suggesting genetic variation in the effect of different families of *R. alectorolophus* on the hosts (i.e. their virulence). Genetic variation in parasite virulence has been found for *R. angustifolius* families grown with two hosts (Ahonen *et al*. 2006), among populations of *R. angustifolius* grown with *A. capillaris* (Mutikainen *et al*. 2000), and for *R. alectorolophus* grown with various host plants (Sandner and Matthies 2017). In contrast, there was no effect of population identity on the virulence of *R. angustifolius* or *R. minor* grown with *Hordeum vulgare* (Rowntree *et al*. 2011).

The effect of the parasite on total productivity also depended on the specific combination of host treatment and parasite family. This indicates that the success of using hemiparasites to reduce grassland productivity (Ameloot *et al*. 2005), promote plant diversity (Davies *et al*. 1997; Bullock and Pywell 2005), increase invertebrate abundance (Hartley *et al*. 2015) and control invasive plants (Těšitel *et al*. 2020) will depend on the genetic diversity of the parasite. Previous research already found that genetic diversity appeared to promote establishment of the related parasite *R. minor* in a high species environment (Rowntree and Craig 2019).

In this study, parasite families that across all host species produced the highest biomass tended to be associated with the smallest hosts, suggesting that they have the strongest negative effects on the hosts. There may thus be selection for increased negative effects on hosts. This is in contrast to the optimum degree of virulence and the prudent rather than maximum resource extraction predicted by the model of Hautier *et al*. (2010) for hemiparasites. However, these authors also cautioned that if several parasite individuals parasitize the same host individual, maximum resource extraction would be favoured (see also Regoes *et al*. 2000; Rigaud *et al*. 2010). Maximum use of host resources by hemiparasites that strongly reduces host growth might also be selected because the hosts are not only an important source of nutrients, carbon and water for the hemiparasites (Těšitel *et al*. 2011), but also competitors for light (Matthies 1995).

The biomass of the parasite and that of the grasses (both *Lolium* and *Dactylis*) grown in the same pot were significantly negatively correlated. This could indicate that large parasites suppressed hosts more strongly than small ones, but also that large host individuals were stronger competitors and prevented strong growth of the parasites. However, similarly strong negative correlations between parasite and host biomass were not found for the other host treatments indicating no clear relationships between resource extraction and parasite vigour. In the case of *Medicago* and *Trifolium* this may have been due to a tolerance of parasitism, as these species are good hosts for *R. alectorolophus*, but little damaged by parasitism (Matthies 2021).

Surprisingly, there was a significant positive relationship between the size of parasites and *Sinapis* individuals grown in the same pot. As *Sinapis* was clearly an unsuitable host one would have expected that the negative effects of the *Sinapis* plants on *R. alectorolophus* due to competition for light and nutrients would increase with their size. A possible explanation for the positive correlation is that the parasites may nevertheless have obtained some resources by parasitism and this benefit may have been positively related to individual host size.

## Conclusions

We found significant variation among seed families from a population of the hemiparasite *R. alectorolophus* in their performance with different hosts. This indicates evolutionary potential and could provide the basis for specialization to different host species. However, absence of trade-offs between the performance with different hosts support the notion that hemiparasites, like many herbivorous insects (García-Robledo and Horvitz 2011), are not constrained in their use of different host species by trade-offs in performance. Together with the strong spatial and temporal variation in host species availability and gene flow, the lack of trade-offs could explain the absence of host specialization within the parasite population (Rigaud *et al*. 2010). Increased damage to hosts (virulence) tended to be positively correlated with parasite fitness in *R. alectorolophus*, suggesting selection for maximum resource extraction from hosts. The variation among seed families of *R. alectorolophus* in their effect on different hosts highlights the importance of the genetic diversity of hemiparasites for their effects on the community structure and diversity of grasslands.

## Supplementary Material

plac063_suppl_Supplementary_Material

plac063_suppl_Supplementary_Data

## Data Availability

Data are available as **Supporting Information**.

## Notes

**Natural History and Conservation. Chief Editor:** F. Xavier Picó
