# Supplementary material for: Performance of a parasitic plant and its effects on hosts depends on the interactions between parasite seed family and host species
Source: AoB Plants. 2022 Dec 15;15(2):plac063. doi: 10.1093/aobpla/plac063 (PMC9893871; doi:10.1093/aobpla/plac063)
Supplement: plac063_suppl_Supplementary_Material [file plac063_suppl_supplementary_material.docx]

**SUPPORTING INFORMATION**

Authors: Belén Moncalvillo, Diethart Matthies

Study: Performance of a parasitic plant and its effects on hosts depend on the interactions between parasite seed family and host species

**Supporting Information Table S1**

**Table S1** Effect of host species (H), maternal family (F), parasite biomass (B) and their interactions on traits of the hemiparasite *R. alectorolophus.* p < 0.05 are in bold face.

| Trait | Biomass | |  | Family | |  | Host | |  | F x H | |  | F x B | |  | H x B | |  | F x H x B | |
| --- | --- | --- | --- | --- | --- | --- | --- | --- | --- | --- | --- | --- | --- | --- | --- | --- | --- | --- | --- | --- |
|  | F | p |  | F | p |  | F | p |  | F | p |  | F | p |  | F | p |  | F | p |
| Height | 3782.9 | **<0.001** |  | 5.7 | **<0.001** |  | 7.6 | **<0.001** |  | 2.0 | **0.004** |  | 1.4 | 0.224 |  | 4.5 | **<0.001** |  | 1.0 | 0.426 |
| Length of vegetative part of stem | 257.5 | **<0.001** |  | 6.4 | **<0.001** |  | 8.0 | **<0.001** |  | 2.1 | **<0.001** |  | 1.1 | 0.353 |  | 3.8 | **0.004** |  | 1.0 | 0.436 |
| Total branch length (log) | 3401.1 | **<0.001** |  | 6.5 | **<0.001** |  | 8.2 | **<0.001** |  | 1.2 | 0.235 |  | 1.3 | 0.228 |  | 5.2 | **<0.001** |  | 1.3 | 0.137 |
| Number of vegetative nodes | 20.8 | **0.003** |  | 56.0 | **<0.001** |  | 2.4 | **0.048** |  | 1.4 | 0.053 |  | 0.4 | 0.916 |  | 0.2 | 0.969 |  | 0.9 | 0.600 |
| Length of first five internodes (log) | 218.7 | **<0.001** |  | 12.0 | **<0.001** |  | 8.5 | **<0.001** |  | 1.1 | 0.310 |  | 0.5 | 0.842 |  | 1.1 | 0.392 |  | 0.7 | 0.911 |
| Days until flowering | 119.7 | **<0.001** |  | 30.2 | **<0.001** |  | 2.2 | 0.067 |  | 1.9 | **0.001** |  | 2.8 | **0.008** |  | 1.1 | 0.361 |  | 1.2 | 0.170 |
| Number of flowers at main inflorescence (log) | 1364.5 | **<0.001** |  | 7.2 | **<0.001** |  | 9.8 | **<0.001** |  | 1.8 | **0.004** |  | 2.8 | **0.008** |  | 3.8 | **0.004** |  | 1.0 | 0.526 |
| Number of flowers (log) | 1548.7 | **<0.001** |  | 3.4 | **0.002** |  | 10.7 | **<0.001** |  | 2.1 | **<0.001** |  | 2.9 | **0.007** |  | 6.7 | **<0.001** |  | 1.2 | 0.198 |
| Flower length | 140.8 | **<0.001** |  | 4.2 | **<0.001** |  | 2.9 | **0.020** |  | 1.1 | 0.385 |  | 2.4 | **0.020** |  | 0.7 | 0.667 |  | 2.1 | **<0.001** |
| Diameter of ripe fruit | 515.9 | **<0.001** |  | 5.0 | **<0.001** |  | 4.1 | **0.002** |  | 2.1 | **<0.001** |  | 1.6 | 0.127 |  | 0.4 | 0.877 |  | 1.3 | 0.126 |
| Leaf chlorophyll content | 146.8 | **<0.001** |  | 8.6 | **<0.001** |  | 28.0 | **<0.001** |  | 2.2 | **<0.001** |  | 2.4 | **0.021** |  | 2.2 | 0.058 |  | 1.0 | 0.501 |
| Length of longest leaf | 6687.9 | **<0.001** |  | 6.5 | **<0.001** |  | 14.9 | **<0.001** |  | 1.5 | **0.038** |  | 0.7 | 0.632 |  | 3.2 | **0.012** |  | 0.9 | 0.663 |
| Width of longest leaf | 1103.6 | **<0.001** |  | 16.0 | **<0.001** |  | 18.5 | **<0.001** |  | 1.4 | 0.069 |  | 3.0 | **0.005** |  | 1.7 | 0.143 |  | 1.0 | 0.508 |

**Supporting Information Table S2**

**Table S2** Trait means for the different parasite maternal families. Different letters in the exponent denote differences between families (Tukey-test, P < 0.05).

| Trait | Parasite seed family | | | | | | | | |
| --- | --- | --- | --- | --- | --- | --- | --- | --- | --- |
|  | Fam1 | Fam2 | Fam3 | Fam4 | Fam5 | Fam6 | Fam7 | Fam8 |  |
| Parasite biomass (mg) ^a^ | 151.4^a^ | 288.4^b^ | 295.1^b^ | 295.1^b^ | 316.2^bc^ | 371.5^bc^ | 407.4^bc^ | 446.7^c^ |  |
| Height (cm) | 23.5^a^ | 28.2b^c^ | 26.6^ab^ | 28.00^bc^ | 31.1^cd^ | 28.2^bc^ | 33.4^d^ | 30.9^cd^ |  |
| Length of vegetative part of stem (cm) | 19.4^a^ | 21.4^ab^ | 20.7^ab^ | 22.4^b^ | 23.4^b^ | 21.3^ab^ | 26.7^b^ | 23.4^b^ |  |
| Total branch length (cm) ^a^ | 20.6^a^ | 25.2^ab^ | 26.0^bc^ | 29.2^bcd^ | 30.3^bcd^ | 27.2^bc^ | 231.7^cd^ | 33.4^d^ |  |
| Number of vegetative nodes | 8.5^b^ | 8.9^bc^ | 7.6^a^ | 7.7^a^ | 9.4^c^ | 8.6^b^ | 11.1^d^ | 8.9^b^ |  |
| Length of first five internodes (cm) ^a^ | 8.3^a^ | 7.5^a^ | 9.9^bc^ | 10.3^c^ | 7.4^a^ | 8.5^ab^ | 7.6^a^ | 8.5^a^ |  |
| Days until flowering | 52.2^c^ | 51.0^cd^ | 46.7^a^ | 49.4^bc^ | 52.4^d^ | 49.0^b^ | 52.4^d^ | 47.6^ab^ |  |
| Number of flowers ^a^ | 4.1^a^ | 6.6b^c^ | 5.7^b^ | 6.2^bc^ | 7.1^bc^ | 7.8^c^ | 7.5^bc^ | 7.8^c^ |  |
| Number of flowers at main inflorescence ^a^ | 3.9^a^ | 6.3^bc^ | 5.6^b^ | 5.5^b^ | 6.6^bc^ | 7.5^c^ | 6.71^bc^ | 6.6^bc^ |  |
| Flower length (mm) | 18.5^ab^ | 19.5^c^ | 18.2^a^ | 18.6^ab^ | 19.2^bc^ | 19.0^abc^ | 19.5^c^ | 19.2^bc^ |  |
| Diameter of ripe fruit (mm) | 10.5^a^ | 11.9^ab^ | 12.2^b^ | 11.4^ab^ | 11.4^ab^ | 12.1^b^ | 11.8^ab^ | 12.9^b^ |  |
| Leaf chlorophyll content (µg*cm^−2^) | 30.1^ab^ | 29.6^a^ | 29.8^a^ | 29.9^ab^ | 29.4^a^ | 34.5^b^ | 27.3^a^ | 30.2^ab^ |  |
| Length of longest leaf (mm) | 28.3^a^ | 39.4^bc^ | 35.3^b^ | 35.5^b^ | 40.7^c^ | 40.8^c^ | 39.3^bc^ | 42.6^c^ |  |
| Width of longest leaf (mm) | 8.4^a^ | 11.0^bcd^ | 12.1^d^ | 10.5^bc^ | 9.9^ab^ | 11.4^bcd^ | 10.2^b^ | 11.8^cd^ |  |

^a^ geometric means

**Supporting Information Figure S1**


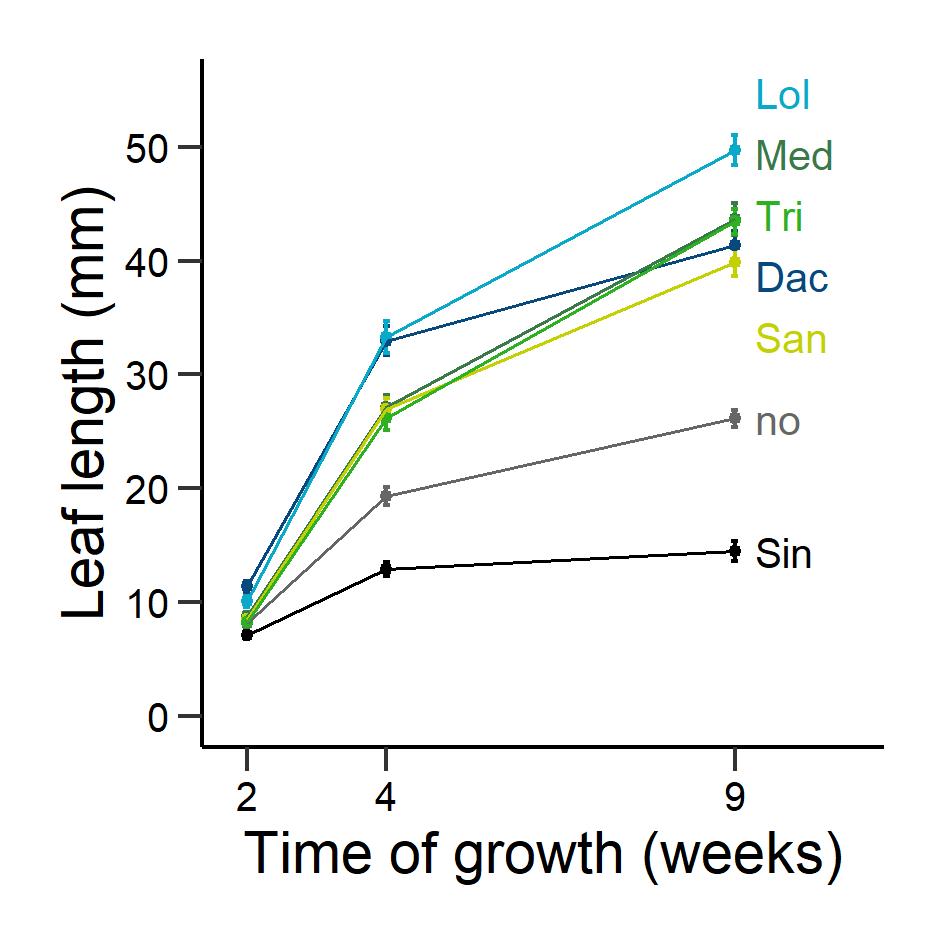


**Figure S1** Growth of *R. alectorolophus* (length of longest leaf) with six different host species and without a host (Lol, *Lolium* *perenne*; Tri, *Trifolium* *repens*; Med, *Medicago* *sativa*; Dac, *Dactylis* *glomerata*; San, *Sanguisorba* *minor*; no, no host; Sin, *Sinapis* *alba*). Vertical lines show ± 1 SE.

**Supporting Information Figure S2**


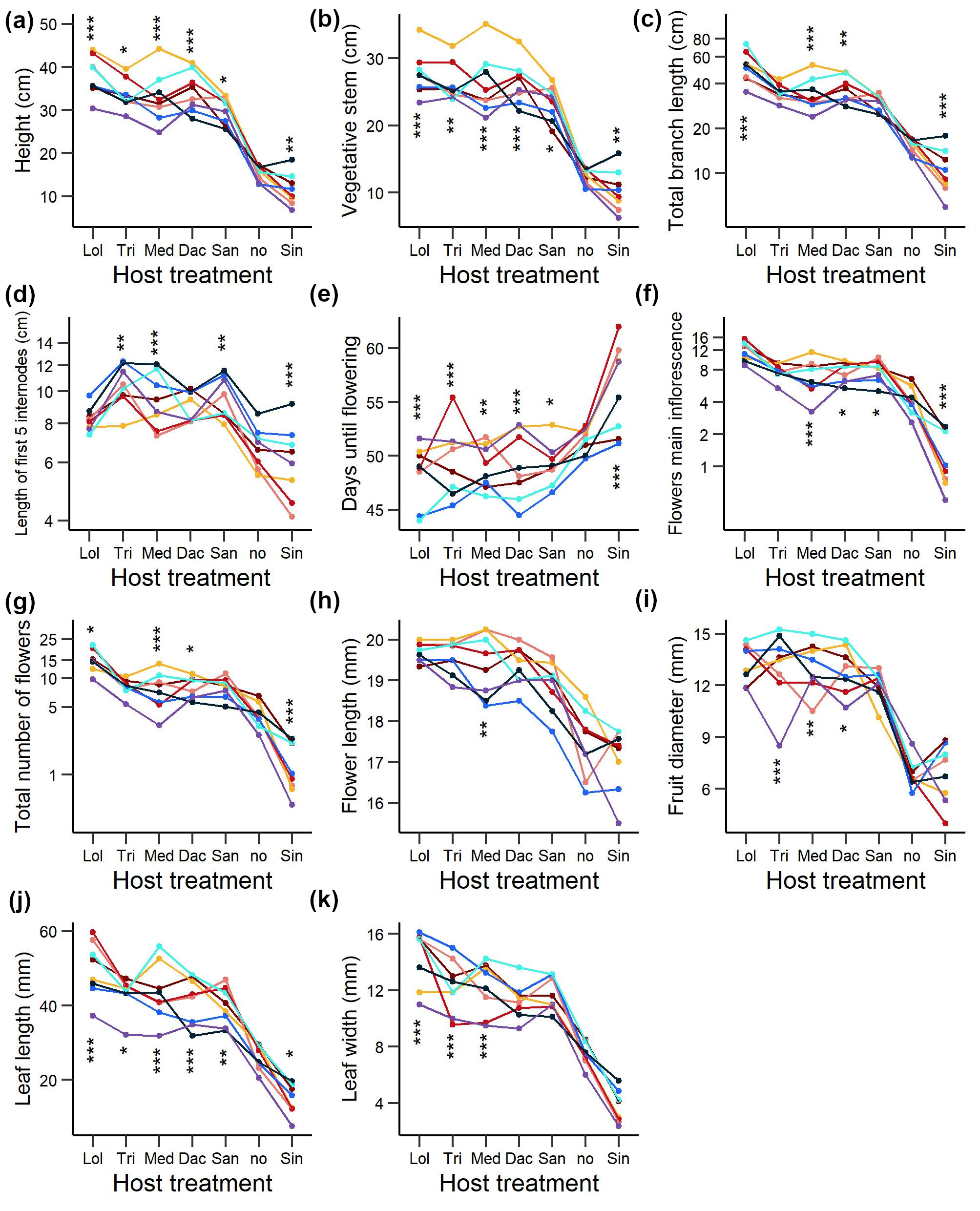


**Figure S2** Reaction norms of several traits of eight maternal families of the parasite *Rhinanthus alectorolophus* in response to the host treatments (six different hosts and no host). (a) Height, (b) length of the vegetative part of the stem, (c) total branch length, (d) length of the first five internodes, (e) days until flowering, (f) number of flowers on the main inflorescence, (g) total number of flowers, (h) length of flowers, (i) diameter of fruits, (j) length of the longest leaf, and (k) width of the longest leaf. The colour of each family is the same as in Fig. 1. Host species are in order of decreasing parasite biomass. Significant differences among families within each host: *, p < 0.05; **, p < 0.01; ***, p < 0.001. For abbreviations of host species names see Fig. S1.

**Supporting Information Figure S3**


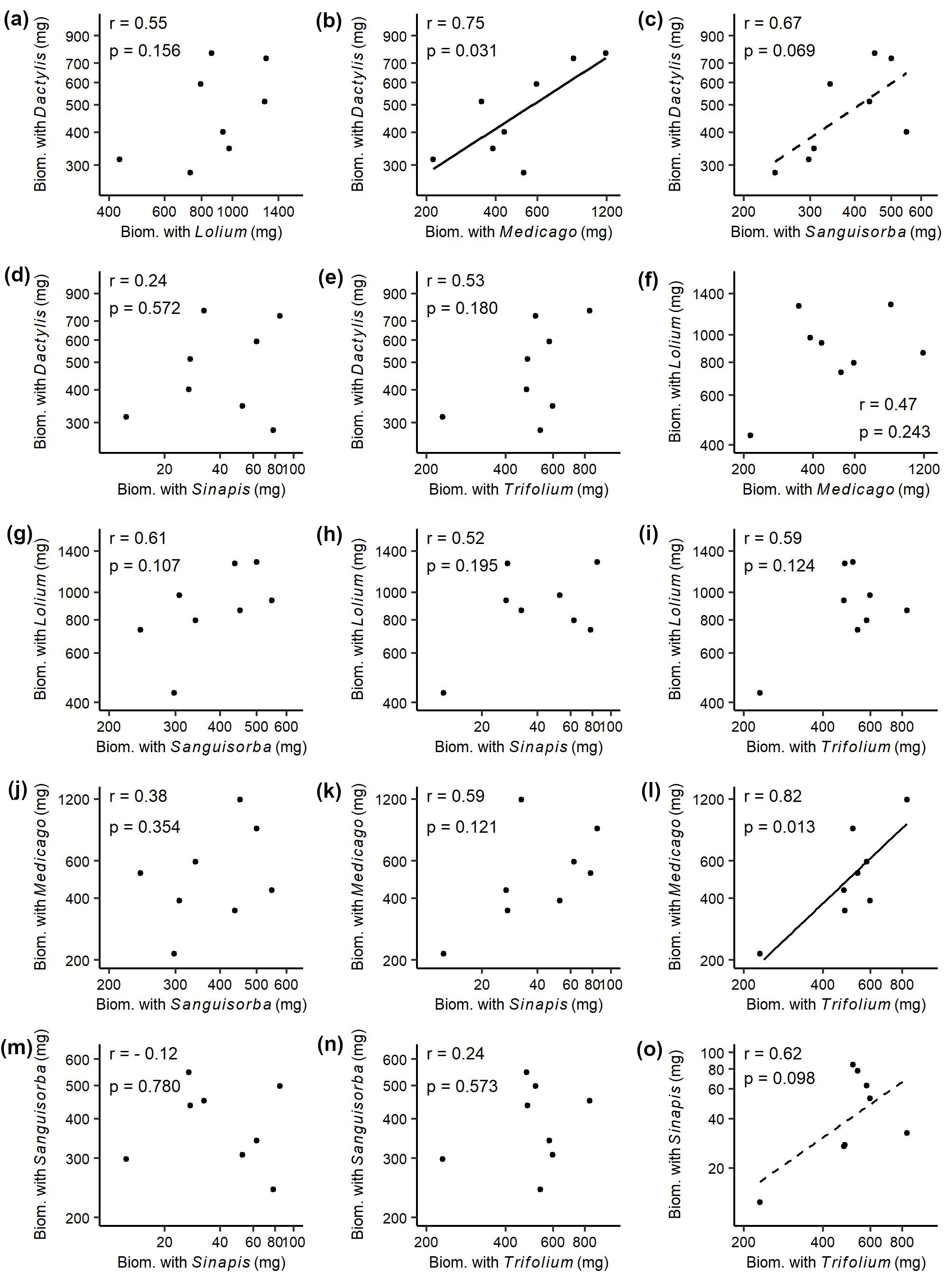


**Figure S3** Pairwise relationships between the mean biomass of individuals of the hemiparasite *R. alectorolophus* belonging to eight different families grown with different hosts. Continuous regression lines are shown if p < 0.05, dashed lines if 0.05 < p < 0.1.

**Supporting Information Figure S4**


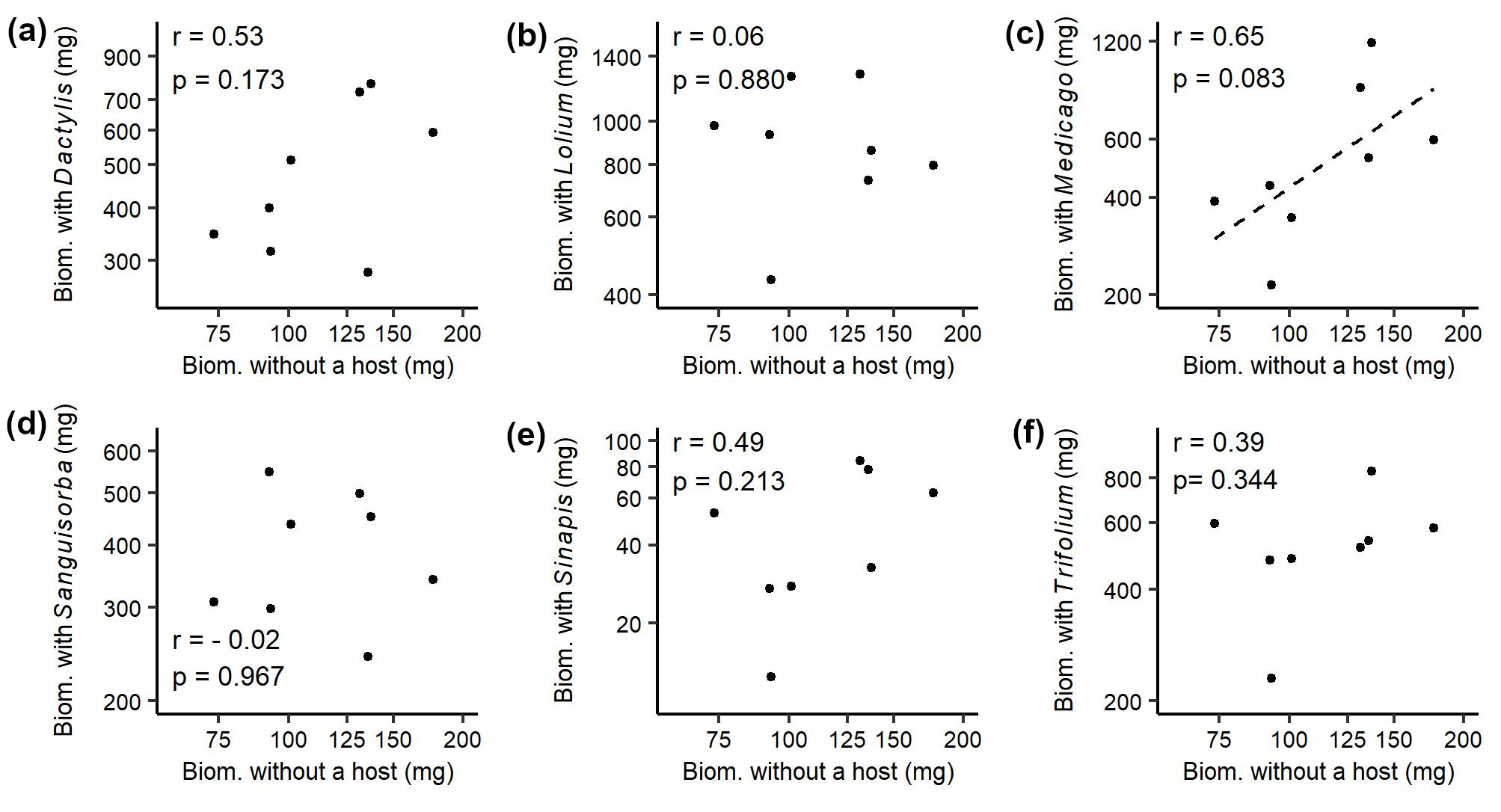


**Figure S4** The relationship between the mean biomass of individuals of the hemiparasite *R. alectorolophus* belonging to eight different families when grown with different hosts and without a host. A dashed regression line is shown if p < 0.1.
